# A Qualitative Study of Living in a Healthy Food Priority Area in One Seattle, WA, Neighborhood

**DOI:** 10.3390/ijerph182212251

**Published:** 2021-11-22

**Authors:** Jenny L. Wool, Lina P. Walkinshaw, Clarence Spigner, Erin K. Thayer, Jessica C. Jones-Smith

**Affiliations:** Department of Health Systems and Population Health, School of Public Health, University of Washington, Seattle, WA 98195, USA; walkinsl@uw.edu (L.P.W.); cspigner@uw.edu (C.S.); erinthayer16@gmail.com (E.K.T.); jjoness@uw.edu (J.C.J.-S.)

**Keywords:** food environment, food access, qualitative research

## Abstract

Policy makers in several major cities have used quantitative data about local food environments to identify neighborhoods with inadequate access to healthy food. We conducted qualitative interviews with residents of a healthy food priority area to assess whether residents’ perceptions of food access were consistent with previous quantitative findings, and to better understand lived experience of food access. We found that proximity to stores, transportation mode, and cost shaped decisions about food shopping. The local food bank played an important role in improving food access. Participants had varied suggestions for ways to improve the neighborhood, both related and unrelated to the food environment.

## 1. Introduction

There are numerous benefits of a healthy diet rich in fruits and vegetables, including lower risk for cardiovascular disease and obesity [1,2], type 2 diabetes [1,3], and all-cause mortality [2]. Despite these known benefits, most U.S. adults do not eat the recommended daily amount of fruits and vegetables [4], and neighborhood disparities in healthy food access may exacerbate this problem. While research suggests an association between better access to large-chain grocery stores and healthier diets [5], lower-income neighborhoods often have fewer chain supermarkets in comparison to middle-income neighborhoods, as do neighborhoods with higher proportions of African American residents in comparison to neighborhoods with higher proportions of White residents [6].

Much of the research on disparities related to neighborhood food environments has focused on food deserts (places without easy access to supermarkets or healthy foods) and physical distance to grocery stores [7]. However, new evidence indicates that food swamps (areas in which unhealthy food retailers outnumber healthy food retailers) may better predict obesity rates [8], and that a relationship potentially exists between fast food restaurant accessibility and prevalence of obesity, with people with obesity more likely to reside in communities with medium or high accessibility of fast food [9].

Recently, a more expansive definition of the food environment includes five dimensions of access in total: availability, accessibility/convenience, affordability, acceptability, and accommodation [7,10]. Availability encompasses the sufficiency of the healthy food supply; accessibility encompasses proximity of and ease of traveling to food stores, with geographic distance and travel time as important measures; affordability encompasses price; acceptability encompasses attitudes about the food environment, including if food meets personal requirements; and finally, accommodation encompasses individuals’ requirements for food and food stores, such as store hours [10].

Building on the expanded definition of the food environment, a study commissioned by the City of Seattle—the 2019 Seattle Healthy Food Availability and Food Bank Network Report—created a new metric using three factors—income, multi-mode travel times to healthy food retailers, and presence of less healthy food retailers (e.g., fast food)—to identify healthy food priority areas, or areas to prioritize for increasing access to healthy, affordable food [7]. Healthy food priority areas are lower-income areas with a large amount of unhealthy food options and long travel times to retailers providing healthy options.

While the 2019 Seattle Healthy Food Availability and Food Bank Network Report quantitatively documented dimensions of the food environment in identified healthy food priority areas in Seattle, we know little about the lived experience of people in these neighborhoods, and if their perceptions of healthy food availability match quantitative accounts of healthy food availability from the 2019 Report. A study of low-income residents in the greater Boston area found that perceived measures of the food environment might be more strongly related to fruit and vegetable intake than objective measures of the food environment [11], underscoring the need for qualitative research to inform quantitative data on food environments.

The purpose of this exploratory study was to understand and seek deeper insights into the lived experience of food access for adults living and/or working in one neighborhood identified as a high priority area, the High Point neighborhood. By lived experience, we mean gaining an understanding of what factors and options influence the perceptions and knowledge of participants [12]. We also sought to understand if perceptions of High Point’s food environment match quantitative accounts of High Point’s food environment from the 2019 Seattle Healthy Food Availability and Food Bank Network Report. Our study complements related research focused on youth perceptions of High Point’s food environment [13]. Additionally, this study sought to understand if members of the community are concerned with increasing healthy food availability and access. Study findings can provide more information on how accurately quantitative measures of the food environment capture lived experience. Findings can also inform interventions and local policies aimed at fostering healthy food environments in healthy food priority areas.

## 2. Methods

### 2.1. Context: The High Point Neighborhood

Beginning in 2000, Seattle’s High Point neighborhood was redeveloped into a mixed-income neighborhood with the creation of new housing designed to be affordable for varied income levels [14]. High Point encompasses 34 city blocks, and is home to Seattle Housing Authority’s “largest family community [14]”. Greater than 25% of people in High Point live below 200% of the federal poverty level [7].

### 2.2. Interview Guide and Demographic Survey Development

We created a draft interview guide by designing interview questions and probes to elicit responses within the five domains of food access: availability, accessibility, affordability, accommodation, and acceptability [10]. For example, we asked “Are there any foods you want to get or purchase, but can’t?” to elicit responses in the domain of either affordability or accommodation, and “From your perspective, how does how close you are to food stores impact the food you eat?” to elicit responses within the domain of accessibility see Table 1 for examples of questions mapped to each domain]. Creation of the draft interview guide was an iterative process informed by the food environment literature and research team members with expertise in qualitative research methods.

To help validate the interview guide, we solicited feedback from community partners at High Point’s local food bank. Discussions ensued between the researchers and staff to determine if questions asked what they purported to ask, and based on feedback, we added a question focused on changes to the neighborhood food environment over time. The final interview guide had eight questions and numerous probes. Midway through data collection, we determined the study guide was not sufficiently gathering enough general information on individuals’ lived experiences in their neighborhood, both related and unrelated to the food environment. For this reason, we added two questions (“How long have you lived in this neighborhood?” and “What has your experience in this neighborhood been like so far?”) to the interview guide for the last five out of fifteen total interviews. This brought the total number of questions in the interview guide to ten.

We also created a nine-question paper demographic survey to be completed post-interview. The survey contained questions about household size, gender, age, income, employment status, marital status, race and ethnicity, and educational attainment.

### 2.3. Sample

We recruited a convenience sample with the help of contacts at two community organizations: the neighborhood food bank and a local organization focused on health, education, and family services. Through conversations with key community stakeholders, we identified these organizations as having meaningful contact with a diverse group of neighborhood residents. We also reached out to other community organizations (e.g., a community housing organization) and posted flyers at the local public library branch.

Eligibility criteria included self-identification as living in High Point or self-identification as an employee in High Point and being over 18 years of age. At the food bank, eligibility criteria additionally included speaking English. At the other community organization, a staff member provided translation for Somali speakers.

### 2.4. Recruitment

At the health, education, and family services organization, staff directly recruited participants by asking people engaged in activities on the days of interviews if they wanted to participate, and by calling community members they thought would be interested and available in advance and asking them if they would like to participate. At the food bank and with the permission of food bank staff, we approached clients in the food bank’s waiting area and determined eligibility and interest. Food bank staff also recruited clients and volunteers by making an announcement asking for participants, and by personally approaching clients who lived in the neighborhood. In total, we recruited 15 participants.

### 2.5. Data Collection

We conducted 15 semi-structured, one-to-one interviews in January 2020 and received verbal consent to conduct and record interviews. Interviews took place on-site in an available room at the health, education, and family services organization, and interviews took place in spare offices at the local food bank. Each participant received an ID number to maintain anonymity. An on-site staff member at the community organization provided translation for three interviews with Somali-speaking participants. Interviews lasted 20 min on average, and each participant received either a $20 gift card or $20 cash. Participants completed the demographic survey following their interviews. While the majority independently completed the survey, three participants had the on-site staff member translate questions and record their answers, and one participant requested their personal helper complete the survey on their behalf. The interviewer also helped participants with limited English literacy if participants requested help. We used participants’ ID numbers to link interviews with demographic surveys.

### 2.6. Data Analysis

We transcribed all interviews and used qualitative analysis software (Dedoose V. 8.3.17, SocioCultural Research Consultants, LLC, Los Angeles, California, USA) for coding and analysis. We analyzed data using a directed content analysis approach [15]. This approach involves developing initial codes based on prior research and theory, and then creating new codes to capture emergent themes that do not fit with the original coding scheme [15]. We created an initial coding structure by developing codes within each of the five domains of food access [10], and later added two additional domains (“Neighborhood level: areas for improvement and changes over participants’ time in neighborhood” and “Other”) to capture emergent themes unrelated to the five domains of food access. We analyzed interview data until no more themes could be uncovered.

After the initial codebook development, the primary coder and secondary coder independently coded one transcript, reconciled differences, and updated the codebook based on discussion. The coders repeated this process with five transcripts, at which point they achieved an inter-rater reliability greater than 80%. Using the finalized codebook, the primary coder single coded remaining interviews and re-coded interviews that were double coded before codebook finalization.

In May 2020, we virtually presented overarching themes to four community stakeholders to corroborate findings.

## 3. Results

Fifteen people participated in this study. On average, participants had lived in the High Point neighborhood for nine years. While participants ranged in age, education level, family size, and marital and employment status, the majority were female (*n* = 13) and reported a household income less than $20,001 (*n* = 12). See Table 2 for complete participant demographics.

Four primary themes emerged from analysis: (1) proximity and transportation mode shaped most food-related decisions; (2) participants perceived healthy food as available but expensive; (3) the neighborhood food bank played a central role in changing neighborhood food access; and (4) participants’ suggestions for ways to improve the neighborhood both included and spanned beyond the food environment.

### 3.1. Theme 1: Proximity and Transportation Mode Shaped Most Food-Related Decisions

For the majority of participants, proximity and transportation mode were the dominant factors influencing where, when, and how frequently to shop for food. While proximity itself was important, it mattered most in relation to participants’ transportation mode.

*“[…] if you got a car, you know, you got major stores around in your proximity. […] Somebody with a vehicle, it’s not as challenging versus somebody who has to depend on like, access to transportation.”*

—Participant 6

*“It’s too far, the shop is too far if you don’t drive.”*

—Participant 13

Consistent with the importance of proximity and transportation mode, participants referenced these factors in response to questions about what makes it hard to get food in the neighborhood and how access has changed over time.

*“Well, being an elderly, you know, it depends on if you have transportation or if you have an aid or if you have a bus pass or, you know, every, it varies on everybody’s situation. So, you know, me, I have [a helper].”*

—Participant 8

A chain drug store played a unique role in the food environment due to its proximity within the neighborhood and accessibility for people without cars. While some neighborhood residents could walk to a local supermarket, a trip there necessitated going down and up a large hill, making the drug store an easier and faster option regardless of transportation mode. Yet, although some participants walked to the drug store to shop for quickly needed items, many expressed disappointment that the store in closest walking distance sold predominately unhealthy options. Two participants felt disappointed the drug store had stopped providing fresh produce.

*“[…] It always involves being able to physically get [food] back to my house because I don’t have a car. That’s going to be true of anyone that uses public transportation. That also ends up meaning that a lot of less healthy food gets eaten because [the drug store] is really close. And they tried an experiment having the fresh fruits and vegetables and they didn’t sell enough and they stopped doing it. […] So if you have a car and if you don’t have a car, it really changes the quality of food you can get if you’re a working person […].”*

—Participant 7

Finally, and as expected, proximity and transportation mode influenced travel times to food stores. Participants who used public transportation emphasized the variability of travel times:

*“It depends on which direction you’re going but the average... you’re talking about, you talk about wait time and actually getting there—could be like roughly anywhere from 20 min to an hour.”*

—Participant 6

*“Sometimes one way you can walk, it takes 40 min to get to store. And then when you come back, a lot of groceries, so cannot walk back. So I had to catch a bus so [the trip there and back] takes two hours, couple of hours […].”*

—Participant 5

Participants also expressed the need to have a cart or bags to help transport groceries, and some discussed needing to plan food shopping around work schedules and other activities.

When participants shopped at stores that were not in close proximity or shopped at multiple stores, they typically did so to save money or to seek out cultural food (e.g., halal meats): “Our culture store is far away from here. But for Americans food is everywhere” (Participant 10).

### 3.2. Theme 2: Participants Perceived Healthy Food as Available but Expensive

Along with proximity and transportation mode, food prices influenced shopping decisions. One participant said that if she is not going to the closest store, sales shape where else she shops:

*“Usually my, my food is purchased at the store, usually [the chain store], that’s the closest store to my house. Otherwise I go to a store where I know something is on sale.”*

—Participant 2

Some participants did not shop at the most proximate and/or within walking distance stores due to price (“we can walk to [a local supermarket] but we cannot afford”), and multiple participants decided where to shop based on sales and traveled farther for better deals. Participants named the ability to buy in bulk or save money via membership as a reason to shop at a membership only retail warehouse. Additionally, while most believed food access in the neighborhood had remained the same or gotten easier over time, three of the four participants who believed access had gotten harder cited the fact that food was more expensive now.

Participants wanted to eat healthy foods but reported high costs, with one-quarter of participants reflecting that more money would enable them or their families to purchase healthier foods (other participants reflected that more money for food would enable greater variety or the ability to occasionally purchase special treats). With the high cost of healthy food, participants appreciated that the neighborhood food bank supplied fresh fruits and vegetables. Although participants were satisfied with the food bank, some noted variable fresh food quality and quantity.

One participant also discussed the intersection of cost and quality at local stores:

*“Sometimes it’s the quality of the food, especially over in [adjacent neighborhood], there isn’t great quality. Like, you can tell there’s like pesticides, or like wax all over the foods so it isn’t like the great, healthier options for healthy foods in itself, or they’ll be way too expensive at a really great grocery store who have great quality. So, just depending on which.”*

—Participant 11

### 3.3. Theme 3: The Neighborhood Food Bank Played a Central Role in Changing Neighborhood Food Access

Nearly half of participants discussed the food bank as a primary reason why access to food in High Point has gotten easier in the time they’ve lived in the neighborhood. These participants ranged in age, education status, and family size. While ten of our fifteen interviews took place at the food bank with food bank volunteers or clients, three of the five participants interviewed at the other local organization also referenced the importance of the food bank, with two of the five volunteering and/or using the food bank and one of the five identifying the food bank as a reason access to food in the High Point community has changed in the time the participant has lived in the neighborhood.

Participants felt the food bank served “hundreds and hundreds” in the community. Participants appreciated the food bank’s central, walkable location within the neighborhood, as well as its varied services, such as providing delivered meals to homebound community members. One participant even suggested that because the neighborhood has the food bank, the city should focus resources on other areas (e.g., education and activities for children) rather than on the food environment.

The majority of participants referenced satisfaction with the food bank’s services, citing availability of dairy-free items and halal chicken as well as appreciation of the food bank’s switch to a grocery store model (a new, but increasingly common design for food banks, which allows clients to come in and “shop” for food) for “allow[ing] people to go to what’s most important to them first” and for “[getting] rid of some of the lack of dignity that sometimes was involved when people had to wait in a line and volunteers were just trying to make them take, make you take things whether you wanted them or not” (Participant 7).

Although the food bank serves large numbers of community members, three participants referenced stigma associated with shopping there:

*“[…] a lot of the community people doesn’t want to come […] some people feel shame, I don’t know, coming to food bank”*

—Participant 5

*“So another thing that folks access is the Food Bank. And I don’t know when that opened up, but I know a lot of, a lot more of our families are starting or have used it, like within the last five years, and there’s sort of a stigma with that, right? If you’re seen at the Food Bank, then it’s like, ah, you know, so and so at the Food Bank, they’re not doing well. So a lot of people tend to avoid it, but, but I feel like the community has opened up a little more for that, you know, for the use of that.”*

—Participant 12

### 3.4. Theme 4: Participants’ Suggestions for Ways to Improve the Neighborhood Both Included and Spanned beyond the Food Environment

Participants reflected on solutions to improve the neighborhood food environment. A majority felt community residents should be part of conversations with City leadership focused on improving food access and availability for the community. Participants’ most common suggestion was increasing the number of food stores in High Point (e.g., “figuring out some way to get a really nice grocery store into this neighborhood would be really, really big”) or creating an epicenter or superstore with both culturally appropriate foods (e.g., Halal meats) and staple items. Participants also discussed wanting to ensure all community members know about and feel comfortable using the food bank and other community organizations.

Participants had many suggestions spanning beyond the food environment for ways to improve the neighborhood. Common suggestions included increasing social services, programs and activities for youth and children, and improving transportation and safety, although three participants discussed safety as something they liked about the neighborhood. Only one participant directly suggested the need for larger-scale, structural change:

*“Well, we can have more stores and you know, and family owned stores and support local, like, you know, businesses and you know, all that jazz. But I think what it really, really comes down to is a structural change. Um, like an equitable change where it’s like, are we really… are we really like giving enough resources to specific communities to strive […].”*

—Participant 12

## 4. Discussion

Most participants identified proximity, transportation, and cost as the most influential factors affecting food-related decisions and access to healthy food. For participants relying on public transportation, travel times to food stores were often variable and long. The majority of participants believed food access in High Point has either remained the same or improved over their time in the neighborhood, in large part due to the presence of the local food bank. While food insecurity did not specifically emerge as a theme, the fact that participants identified the food bank as an important resource may indicate an aspect of food insecurity in the community. Participants’ suggestions for ways to improve food access in the neighborhood included increasing the number of stores and ensuring people know about the food bank and its resources. Participants additionally suggested other areas to focus resources on, such as programs for youth.

Findings were largely consistent with those of a complementary qualitative study among youth living in the same neighborhood [13]. Similar to adults, youth found that cost was the primary factor shaping food-purchasing decisions [13]. Both studies also found transportation to shape food-purchasing decisions. All youth participants’ families used a car to access food, which mitigated the lack of nearby food retailers and led youth to not perceive proximity as an issue for food access, although youth still commented on High Point’s lack of grocery stores and thought accessing food without a car would be difficult [13]. Among adult participants, not everyone had cars, and adults discussed how transportation mode, including having or not having a car, shaped shopping decisions by determining which stores were in proximity. Thus, both youth and adults found transportation to affect shopping decisions.

A central theme in the youth interviews was that families shopped at multiple stores for convenience and to obtain culturally relevant food; while several adults mentioned travelling farther distances or shopping at multiple stores to seek out cultural foods, the importance of culturally relevant foods was not a central theme as in the youth interviews [13]. However, the fact that this topic emerged in both youth and adult interviews underscores its significance. Expanding culturally relevant offerings within the community, either at the food bank or in stores in close proximity to the neighborhood, could be an action with high community impact. Youth also discussed dissatisfaction with school lunch and the importance of the youth voice in conversations around food access [13]. While adults did not discuss these issues, they were not specifically probed on school lunch or the youth voice in food-related conversations.

In sharing study results with community stakeholders, we found that these findings resonated, and findings are largely consistent with those of other qualitative studies in different locations, which have found geographic location [16], transportation [17,18], and cost [16,19] to be important factors shaping shopping decisions and access to healthy food.

This study’s results largely indicate that subjective perceptions of High Point’s food environment largely matched quantitative data from the 2019 Seattle Healthy Food Availability and Food Bank Network report. The 2019 report indicated that High Point had the lowest number of food stores and one of the highest food swamp scores (at the 90th percentile) in the city of Seattle [7], aligning with participants’ experiences of short travel times to convenience stores providing less healthy options (e.g., the chain drug store); similarly, the report indicated that High Point residents had an average one-way travel time of 10 min or more (longer than other Seattle neighborhoods) to the nearest four healthy food establishments [7], aligning with participants’ descriptions of long travel times to healthy food retailers. The finding that subjective perceptions and quantitative data largely matched is in contrast to findings of Gustafson et al., who found differences between objective and perceived measures of food store availability among low-income women in North Carolina [20].

This study’s findings also support the 2019 Seattle Healthy Food Availability and Food Bank Network report’s qualitative data from focus groups conducted at seven Seattle food banks, namely that clients desire a dignified experience, quality food, and expanded hours of operation and other services to improve community access [7].

Although participants did not discuss effects of racism on the five domains of food access, a large body of recent research [21] demonstrates the pervasiveness of structural racism in the food system, which undoubtedly affects food access and availability.

This study elucidated new findings with important ramifications for local policy. Community residents wanted to be involved in decisions about the food environment, and many had other areas they would rather see the city support, such as family programming. This study’s findings suggest that healthy food priority areas might have other pressing needs beyond food access, and that partnering with community members is important for understanding community needs.

Additionally, this study’s findings contribute to current literature on the relative importance of the five domains of food access. Since relatively few studies have focused on the domains of accommodation and accessibility [10], we intentionally asked questions designed to elicit responses within those domains. However, participants did not discuss topics within the domains of acceptability (attitudes about the food environment, including if food meets personal requirements), accommodation (individuals’ requirements for food and food stores, such as store hours of operation or having culturally relevant food), and availability (sufficiency of healthy food supply) to the same extent as topics within the domains of accessibility (proximity/ease of traveling to food stores) and affordability (price of food). Within the domain of acceptability, participants occasionally referenced variable food quality and quantity at the local food bank as not meeting personal standards, and a few brought up wanting to shop at a membership-only retail warehouse because of the option to buy in bulk, but otherwise, participants did not discuss acceptability. Similarly, within the domain of accommodation, only two people discussed a desired change in store hours, although various participants discussed wanting food stores to accommodate healthy diets and culturally appropriate food. For the domain of availability, participants discussed availability of healthy food when prompted, but few continuously or organically brought up topics related to the supply of healthy food in their neighborhood.

While these findings suggest that the domains of accessibility and affordability play the greatest role in influencing food access and food-related decision making, another explanation is that these domains are easier to measure, which is consistent with the fact that the majority of research on the food environment has focused on affordability, availability, and accessibility [10]. Moreover, in sampling from the local food bank, we likely heard from community members with limited budgets for food spending, which might lead to a prioritization of affordability. In the broader community, residents might be more concerned with other domains of food access.

Our data also suggest that within the domain of accommodation, desires for culturally appropriate foods and healthy foods are more pressing than wanting or needing stores to adjust their hours of operation or accept different payment types. When participants shopped at multiple stores or distant stores, they often did so to seek out cultural foods. In contrast, no participants discussed payment as an area in which they desired greater accommodation.

### Limitations

Due to the COVID-19 pandemic, we were unable to present findings in person to the broader community, or to corroborate findings with stakeholders at the local food bank.

While we intentionally asked questions designed to elicit responses about each of the five domains of food access, it is possible some domains had more directive questions than others, which could have inflated the relative importance of these domains. Approach bias and social desirability bias also might have affected responses. Food bank staff were instrumental in recruitment and may have selected participants based on participants’ attitudes towards the food bank. Additionally, two-thirds of interviews took place at the food bank, and participants may have overstated the food bank’s significance to the community and/or their personal satisfaction with the food bank. However, participants who were not interviewed at the food bank also discussed its important role in the community, indicating findings of the food bank’s importance were likely not entirely due to social desirability or approach bias.

Both interviewers (JW and EKT) are white, female, and, at the time of data collection, were Master of Public Health students and non-residents of the neighborhood. The interviewers’ positionality may have influenced interpretation of the data and/or affected participants’ responses.

Study recruitment methods and inclusion criteria had some possible limitations. We recruited from two sites that provide food and/or resources, and in doing so, we potentially missed community members who are struggling with food access but are not using community resources. Study inclusion criteria consisted of self-identification of living or working in the High Point neighborhood, so some participants might not have lived within city-defined neighborhood boundaries. Rather than checking participants’ addresses, we chose self-identification as inclusion criteria to maintain participant privacy and out of recognition that self-identification with a community is perhaps more important than living within city-defined boundaries.

Finally, 12 participants (85.7%) reported household incomes below $20,001. While our predominately low-income sample was consistent with the fact that High Point was identified as a healthy food priority area in part due to higher poverty levels, future research should consider recruiting an economically diverse sample to investigate whether the relative importance of the five domains of food access changes based on income.

## 5. Conclusions

Interviews with members of Seattle’s High Point community largely suggested that perceptions of the food environment matched quantitative accounts. Interviews reflected the importance of proximity to stores, transportation mode, and cost in shaping decisions about where to shop for food, as well at the importance of the local food bank in making food access easier over time. Most felt community residents should be part of conversations focused on improving food access and availability for the community. Participants had varied suggestions for ways to improve the neighborhood, both related and unrelated to the food environment. These findings underscore the importance of including community residents in conversations regarding local policy or interventions related to the food environment, as community members not only want to be included but also may prefer limited resources to be directed to other facets of the community.

Our findings suggest that the domains of accessibility and affordability may play the greatest role in influencing food access and food-related decision making, and within the domain of accommodation, the desire for culturally appropriate foods may be more pressing than wanting or needing adjusted store hours or acceptance of different types of payment.

## Figures and Tables

**Table 1:** Domain Mapping of a Sample of Interview Questions and Probes.

Domain	Questions or Probe
Introductory Questions	Can you tell me about your favorite foods to eat?
	What has your experience in this neighborhood been like?
Availability: sufficiency of the healthy food supply	Can you walk me through a typical day of getting food in your household?
	Do you feel healthy food access is a concern of your community?
Accessibility/Convenience: proximity of and ease of traveling to food stores, with geographic distance and travel time as important measures	From your perspective, how does how close you are to food stores impact the food you eat?
Affordability: price	Can you tell me about any food assistance programs you use?
	Are there any foods you want to get or purchase, but can’t?
Accommodation: individuals’ requirements for food and food stores, such as store hours or culturally relevant food	What makes it hard to get food in your neighborhood?
	Are there any foods you want to get or purchase, but can’t?
Acceptability: attitudes about the food environment, including if food meets personal requirements	City council members are talking about how to improve access to food in your neighborhood. What are some things you would like to see changed?

**Table 2:** Demographic characteristics of participants (N = 15).

Variables	*n* (%)
Number of years living in neighborhood	9.1 (6.5) ^1,2^
Food bank patronage	12 (80)
Household size	3.6 (2.1) ^1^
Gender Female Male	13 (86.7) 2 (13.3)
Age Range 18–34 35–44 45–64 55–64 65+	2 (13.3) 4 (26.7) 2 (13.3) 2 (13.3) 5 (33.3)
Annual Household Income ^2^ <$10,000 $10,001–$20,000 $40,001–$50,000 >$60,000	8 (57.1) 4 (28.6) 1 (7.1) 1 (7.1)
Employment ^3^ Employed Out of work and looking for work Student Retired Unable to work	8 (53.3) 2 (13.3) 1 (6.7) 4 (26.7) 1 (6.7)
Marital Status Married/Domestic partnership Divorced/Separated Single, never married Widowed	5 (33.3) 5 (33.3) 4 (26.7) 1 (6.7)
Education ^2^ High school graduate Some college vocational school College degree Advanced degree Other	2 (13.3) 5 (33.3) 2 (13.3) 2 (13.3) 3 (20)
Race/Ethnicity ^3^ Non-Hispanic White Non-Hispanic Black or African American Non-Hispanic Asian, Native American, American Indian, or Alaska Native ^4^	4 (26.7) 8 (53.3) 3 (20.0)

^1^ Mean and Standard Deviation. ^2^ One non-response. ^3^ Participants could check multiple options. ^4^ We combined categories to preserve anonymity due to small sample size in one of the categories.
